# Dr. Kuan-Teh Jeang (1958–2013): an outstanding scientist, a caring mentor, a role model and leader of the Asian American scientist community -- an eulogy delivered by Paul Liu at NIH on February 8, 2013, with additional modifications

**DOI:** 10.1186/2045-3701-3-14

**Published:** 2013-02-28

**Authors:** Paul Liu, Chou-Zen Giam, Zhi-Ming Zheng

**Affiliations:** 1 National Human Genome Research Institute, NIH, Bethesda, USA; 2 Uniformed Services University of the Health Sciences, Bethesda, USA; 3 National Cancer Institute, NIH, Bethesda, USA

## 

We are here today to remember and celebrate the life of Kuan-Teh Jeang. The 12 days since his passing haven’t made this day any easier, have they? I still feel numb --- as numb as the day I heard the news. I will feel numb, I’m sure, 12 days from now and long after that. Like many of you, I just can’t believe that I can’t call up Teh for advice or, indeed, just call him for no reason at all.

We all are united in our diverse reasons for being here today. Teh was my friend and my colleague. What was Teh to you? He was a superb and insightful scientist. A warm and caring mentor. A highly successful journal editor. A role model. A tireless and inspiring leader. An advocate. A change agent. A 54-year-old child at heart. A father. A husband. A son-in-law.

With Teh being so many things to so many of us, allow me to say a few words about Teh’s life, work, and personality.

Teh was born in Taiwan, but spent his childhood in Libya between ages 5 and 12. He came to the United States in 1970 as a teenager. A highly gifted student, Teh entered MIT as an undergraduate at age 16. Two years later he joined an accelerated program at Hopkins, receiving both MD and PhD degrees at the age of 25. Since 1985, he had been a scientist at NIH. He was first a postdoctoral fellow in George Khoury’s lab, and then set up his own group in 1988. He was tenured in 1993, and had been a Section Chief in the Laboratory of Molecular Microbiology in NIAID.

Teh was well known in the virology field, especially for his groundbreaking studies on HIV and HTLV-1 viruses. His contributions were numerous and seminal. It would probably take a whole day to describe them all. He addressed fundamental questions revolving mostly around the themes of HIV replication and pathogenesis, and how HTLV-1 transforms cells through its oncoproteins. Being a curious and inquisitive scientist, he sometimes forayed into other areas as well, that often led to exciting surprises.

Teh’s research efforts in the 80’s and 90’s focused on understanding the mechanisms of action of Tat and Tax, the transcriptional activators of HIV and HTLV-1. His works during that period harken back to the time when very little is known about how eukaryotic genes are regulated by transcriptional enhancers and promoters. He, like many other virologists, recognized how the simple and elegant designs of retroviruses such as HIV and HTLV-1 may be exploited to gain insights into the inner working of eukaryotic transcription.

His lab was the first to demonstrate in 1989 that the nascent TAR RNA in the 5′ end of the HIV transcript interacts with Tat. This finding heralded the identification of TAR-interacting cellular factors, now known to be cyclin T1 and CDK9, or collectively called pTEFb, to promote transcriptional elongation of viral mRNA transcript. The pausing of RNA Pol II at the 5′ end of the nascent mRNA transcript waiting for signals to complete mRNA synthesis is now recognized to be a major regulatory mechanism of eukaryotic mRNA transcription. In 1991, Teh’s lab identified a cellular TAR RNA-binding protein (TRBP), which had since been demonstrated to be an important component of Dicer-mediated miRNA/siRNA maturation pathway.

This was then followed by discoveries that the HTLV-1 oncoprotein, Tax, causes genomic instability by interfering with DNA repair and mitotic functions. These activities of Tax impact on cell transformation and leukemia development. His lab was also the first to discover an interacting partner of Tax, he called TaxBP1, that has been gaining in importance and has been shown recently to be a subunit of the A20-containing enzyme complex responsible for turning off the signaling cascade that leads to lysine 63 polyubiquitination and IKK activation.

His interest in genomic instability and cancer induced by HTLV-1 Tax had led serendipitously to two important discoveries. The first was MAD1, a core component of mitotic checkpoint, discovered in his lab in 1998. The other one was the recognition that the accumulation of a nuclear envelope protein SUN1 causes progeria in 2012. Many of you had heard his beautiful talk on this second subject in the 2012 Khoury Lecture at NIH.

Teh had been incredibly productive, having published more than 300 papers and edited six books. The importance and the impact of his papers can be measured by the number of citations of his papers, which is 19,513 with an h-index of 75, according to the web site of Google Scholar Citations (http://scholar.google.com/citations?user=uiPIDmsAAAAJ&hl=en&oi=ao). These are both exceptionally high numbers.

Teh was awarded for his achievements by elections to prestigious societies such as ASCI, AAP, AAAS (Fellow), and Academia Sinica. He was a recipient of many awards and lectureships. Among them I think what Teh treasured the most was the 2012 Khoury Lecture at the NIH, in October. The Khoury Lecture series was initiated by Teh to honor his former mentor, Dr. George Khoury, an outstanding NIH scientist who also died in his prime. Previous Khoury lecturers include many luminaries in biomedical research, such as David Baltimore, Phil Sharp, Robert Tjian, Bert Vogelstein, and Robert Weinberg. The Khoury Lecture Teh delivered was one of the best lectures I have ever attended. He was masterful, eloquent, commanding, and explained complicated and specialized research topics in ways that could be easily understood by others outside of his field.

Teh was actively involved in scientific publishing. He was an associate editor of Cancer Research, and was on the editorial board of numerous other scientific journals, including the *Journal of Virology* and *JBC*. Teh was extremely proud of the journal *Retrovirology*, which he shepherded and had been its Editor-in-Chief since its inception. As I learned from retrovirologists close to Teh, each year they would receive an e-mail from him inviting them to submit manuscripts, to help with manuscript reviews, and to write review articles. He was most proud of the ever-rising impact factor of *Retrovirology*, now at 6.47, the highest among virology journals. For his efforts, Teh was named BioMed Central’s Open Access “Editor of the Year” in 2010.

Teh was a phenomenal mentor. He worked tirelessly for his students and postdocs, but also demanded the best of them. From what I heard, at the biennial HTLV-1 meetings, there would always be a gathering of the alumni of the Teh lab in a nice restaurant, and the room would be full of laughter, and the energy in the room would invariably be highly charged. Needless to say, he always footed the bill. His current and former students and postdocs adored him. Postdocs in his lab landed good jobs afterwards. That is because he would go out of his way to advance their careers, getting them great publications, writing them strong letters, and making phone calls. Teh’s warm and caring supports also extended to many friends and colleagues outside his lab. He would nominate them for awards and elections to prestigious positions. He would help them with their experiments and publications. He had countless collaborators (myself included). He would extend a helping hand to friends who need personal assistance as well. Here is a story a good friend of Teh told me: In 1999, this friend’s wife was diagnosed with a type of cancer that was relatively rare in the US. It was Teh, through his large network of friends, arranged for them to see a renowned expert on this type of cancer, who eventually cured this friend’s wife of the disease. This is just one of many examples of Teh’s kindness.

Outside of the lab --- hmmm, maybe inside the lab, too, Teh was a college basketball fanatic. I was told that at the time of March Madness, he would be setting up a pool with charts and score boards to see who would eventually win. (But I believe that he did not collect any money at NIH.) Teh also had many passions other than college basketball. In fact, he had strong opinions about many things. I did say “change agent” earlier. However, Teh was not the type who would force his ideas on you, even though he thought he was right. But he would keep trying, however subtle, until you turned around. You may not always agree with him initially, but after listening to his articulations, which were almost always eloquent and insightful, you would often begin to think about the issues from a different perspective. That often led you to re-examine the issues. The “salesmanship” or the “persuader” side of Teh had served him well for his causes. Look at how many people are in this room and how much stronger the SCBA has become.

Teh was a long time member of the SCBA (which stands for Society of Chinese Bioscientists in America) and served as the chapter president for the Baltimore-DC chapter in 2005. In 2009, he was elected as the President of SCBA for a two-year term (2010–2011). During his tenure, he converted the budget of the Society from deficit to surplus and organized a highly successful biennial meeting in 2011. He tirelessly promoted the Society and recruited many new members during his two years of service. Moreover, Teh conceived and guided the creation of a new journal, *Cell & Bioscience*, as the first official journal of the SCBA. This new journal was perhaps the biggest achievement of Teh’s tenure as the SCBA President. The journal has been very successful in its first two years of existence. It was accepted for tracking impact factors by ISI starting with the first published paper, a fate normally given only to publishers such as *Cell, Nature* and *Science* series. Teh was instrumental for this. The journal should serve the SCBA well in the future, both scientifically and financially.

To many friends and colleagues at the NIH, Teh was best known for his passionate and undeterred calls for fair representation of Asian Americans in leadership positions. Teh was a pioneer in raising the issue of “glass ceiling” for Asian-American scientists. I still remember the first phone call I received from him, in late 2005, shortly after the publication of the famous *Science* article, in which Teh was interviewed extensively on this issue (*Science* 310:606, 2005). Initially I was skeptical, since I thought I had never experienced any discrimination myself, especially not at the NIH. But the data Teh showed me were striking. The data described in the *Science* article showed a clear bottom heavy/top light pattern for Asian scientists at NIH, with large numbers of Asian trainees but very few at leadership positions. Why did the numbers exist and how could we change the trend? Teh was the first to ask these questions at the NIH and had called for a change for many years.

In preparing this eulogy, I discovered a *Science* article by Ron R. Hoy in 1993, on the topic of “A ‘Model Minority’ Speaks Out on Cultural Shyness” (*Science* 262:1117, 1993). In this article Hoy argued that one reason for the numbers is the perceived perpetual “foreignness” of Asians. No matter how long you have been in this country, even if you were born here, you are considered a foreigner. One example in the article was that of a 23-year-old graduate student named Kuan-Teh Jeang, who was preparing two scientific talks at a Cold Spring Harbor Meeting in 1982 and who learned that a prominent virologist had asked a co-worker whether this Jeang guy spoke English. Teh quipped that the stereotyped perception was: “If they can’t pronounce your name, then you probably can’t pronounce theirs either.” Another widespread stereotype of Asians is that they prefer and are content to be just workers and not leaders. These views of Asians likely contributed to the “glass ceiling” or so-called “bamboo ceiling” placed over Asian Americans at the NIH and at other academic institutions.

Teh had been very outspoken on this issue. He would talk to anyone at the NIH who would listen. He tried to motivate his Asian-American colleagues to work with him on this issue (and had been very successful in doing so). He would persuade and enlist helps from his colleagues of other ethnic groups. He was very active on the NIH Asian-Pacific Islander Advisory Committee, which advises the Director of OEODM on issues related to Asian American employees. He met with top leaders of NIH to discuss this issue on multiple occasions. He even testified before the EEOC Commissioners in 2008.

Teh liked to compare the situation of Asian scientists with African-American football players. In fact he mentioned this comparison at the SCBA Baltimore-DC chapter holiday dinner two months ago, the last official SCBA gathering he attended. He pointed out that even though there were many black players in the NFL, very few, if any, were head coaches or quarterbacks 20 years ago. Now there are many of them. Such changes didn’t happen overnight, and would not have happened without conscious and serious efforts from both sides – the players and the league management. Teh had been a champion for increased representation of Asian-American scientists at leadership positions at the NIH. Even though we still have a long way to go, I am happy to report that we have made progress. Asian scientists now occupy 8% of branch/lab chief positions at NIH, up from <5% in 2005. We need to thank Teh for this.

In closing I would like to quote a couple of comments that Teh made. First, Teh said during his EEOC testimony in 2008: “How would I characterize the state of Asian-American scientists at the NIH today? … My short answer … is that we have come a long way, but we still have miles to go before we can rest.”

In his annual Holiday Greetings to me and many of you last December, he repeated a similar line: “…. I can say that we have come a long way. We may not have received all the answers yet, but at least it is my impression that we are asking the right questions and others are paying attention.”

I also would like to quote the German writer Goethe, who said “A useless life is an early death.” If the converse is true, by Goethe’s standards, Teh lived a long, long life… The Chinese historian and scholar Si Ma Qian also said: “. Teh has served the community tirelessly and unselfishly, so he died . Teh will continue to live in the ideals he has instilled in his children, in his postdocs, and --- I don’t hesitate to say --- in me and surely in many of you.

We will miss you, Teh. Please rest peacefully in heaven. Those of us you inspired will continue the quest you began many years ago and will not rest until we reach the goals you dedicated your life for.

